# SigFuge: single gene clustering of RNA-seq reveals differential isoform usage among cancer samples

**DOI:** 10.1093/nar/gku521

**Published:** 2014-07-16

**Authors:** Patrick K. Kimes, Christopher R. Cabanski, Matthew D. Wilkerson, Ni Zhao, Amy R. Johnson, Charles M. Perou, Liza Makowski, Christopher A. Maher, Yufeng Liu, J.S. Marron, D. Neil Hayes

**Affiliations:** 1 Department of Statistics and Operations Research, University of North Carolina at Chapel Hill, Chapel Hill, NC 27599, USA; 2 The Genome Institute, Washington University School of Medicine, St. Louis, MO 63108, USA; 3 Division of Oncology, Department of Medicine, Washington University School of Medicine, St. Louis, MO 63108, USA; 4 Lineberger Comprehensive Cancer Center, University of North Carolina at Chapel Hill, Chapel Hill, NC 27599, USA; 5 Department of Genetics, University of North Carolina at Chapel Hill, Chapel Hill, NC 27599, USA; 6 Fred Hutchinson Cancer Research Center, Seattle, WA 98109, USA; 7 Department of Nutrition, University of North Carolina at Chapel Hill, Chapel Hill, NC 27599, USA; 8 Carolina Center for Genome Sciences, University of North Carolina at Chapel Hill, Chapel Hill, NC 27599, USA; 9 Department of Biostatistics, University of North Carolina at Chapel Hill, Chapel Hill, NC 27599, USA; 10 Multidisciplinary Thoracic Oncology Program, Division of Medical Oncology, Department of Internal Medicine, University of North Carolina at Chapel Hill, Chapel Hill, NC 27599, USA

## Abstract

High-throughput sequencing technologies, including RNA-seq, have made it possible to move beyond gene expression analysis to study transcriptional events including alternative splicing and gene fusions. Furthermore, recent studies in cancer have suggested the importance of identifying transcriptionally altered loci as biomarkers for improved prognosis and therapy. While many statistical methods have been proposed for identifying novel transcriptional events with RNA-seq, nearly all rely on contrasting known classes of samples, such as tumor and normal. Few tools exist for the unsupervised discovery of such events without class labels. In this paper, we present SigFuge for identifying genomic loci exhibiting differential transcription patterns across many RNA-seq samples. SigFuge combines clustering with hypothesis testing to identify genes exhibiting alternative splicing, or differences in isoform expression. We apply SigFuge to RNA-seq cohorts of 177 lung and 279 head and neck squamous cell carcinoma samples from the Cancer Genome Atlas, and identify several cases of differential isoform usage including *CDKN2A*, a tumor suppressor gene known to be inactivated in a majority of lung squamous cell tumors. By not restricting attention to known sample stratifications, SigFuge offers a novel approach to unsupervised screening of genetic loci across RNA-seq cohorts. SigFuge is available as an R package through Bioconductor.

## INTRODUCTION

Today, massively parallel next-generation sequencing platforms offer unbiased analysis of transcriptomes at higher accuracy and resolution than microarrays (1). Beyond measuring expression levels, transcriptome sequencing (RNA-seq) can be used to discover novel transcriptional events such as splicing patterns (2), alternative untranslated region usage (3) and gene fusions (4). With the rise of platforms capable of producing large-scale genomic datasets, unsupervised methods have played an increasingly major role in the analysis of such data. Arguably, among unsupervised approaches, clustering methods have had the most visible impact on the field. Briefly, clustering methods provide the ability to discover grouping structure within datasets without knowledge of *a priori* classes. In past studies, hierarchical clustering has been applied to microarray expression profiles to identify clinically relevant subclasses of cancers and other diseases (5–7). As such, extensions of these approaches to modern sequencing platforms could potentially be used to identify unrecognized structure with applications to a variety of problems.

An emerging area of genomic research is the identification of alternative splicing events, i.e. when pre-mRNAs are spliced in different ways to produce distinct isoforms, ultimately encoding for different proteins (8). Recent estimates suggest that most human genes are alternatively spliced, with most alternative exons showing tissue-specific regulation (9). Further, alternative splicing and isoform selection have been implicated as determinants of cell type and specificity (10). Within individual samples, multiple isoforms are often simultaneously expressed at a single gene. Therefore, identifying differential isoform usage, where multiple isoforms of a single gene are expressed, but at different proportions between groups of samples, may provide insight into the functional consequences of a disease.

Throughout this paper, we will refer to a region of the genome to which a single gene has been annotated as a *locus*. Using RNA-seq, expression at a single gene, or locus, can now be measured at each base-position along the length of the transcript, making the technology sensitive to isoform level changes in expression. Thus, genome-wide discovery of alternate isoform usage is an opportunity afforded by RNA-seq, beyond what was possible using gene expression arrays. Our approach is motivated by the desire to realize the full potential of RNA-seq data.

Several methods have been suggested for the detection of alternative splicing or isoform differences in supervised settings, e.g. in a tumor versus normal comparison, including Cuffdiff 2 (11), DEXSeq (12) and DiffSplice (13). However, differences in isoform usage may not always correspond to known class labels, e.g. differential usage may exist between subsets of a single tissue type. As an example, the usage of a novel *CDH3* splice variant was reported in only a subset (8/20) of adenocarcinoma tumors relative to normal (14). In this case, the differential signal may become lost within the larger tumor versus normal comparison, and further, the subtype behavior completely missed. Currently, it is not clear how to identify differential isoform usage when the appropriate stratification of samples is unknown.

To address these problems, unsupervised approaches, including clustering, have complemented supervised analyses in genomics. Earlier on, approaches to whole-genome clustering, i.e. clustering by gene expression across all loci, were proposed for RNA-seq data (15). More recently, SIBER (16) and DEXUS (17) have been proposed for clustering samples at the single gene level, i.e. clustering at each gene separately, to discover novel subpopulations exhibiting differential expression at individual loci. However, these methods were not specifically designed to detect differences in isoform usage as they only consider gene-level expression.

In order to detect subsets, or clusters, of RNA-seq samples with alternative forms or patterns of isoform usage, we have developed SigFuge (SIGnificant Forms Using per-base Gene Expression). SigFuge aims to identify clusters that express isoforms from a single gene locus at differing proportions. That is, we seek to identify clusters with differing isoform preferences at the level of single genes. This is possible because SigFuge uses expression levels at each base-position across a gene locus. Briefly, for each locus, the approach first requires filtering out lowly expressed samples. Then, among the remaining samples, SigFuge normalizes expression at the base-pair level. This normalization allows SigFuge to emphasize expression differences occurring throughout a segment of the gene, e.g. exon-level differences, while ignoring differences occurring across the entire gene, e.g. whole gene gain/loss, which methods such as SIBER and DEXUS aim to identify. Next, the samples are clustered into two subpopulations by the normalized base-pair level expression, and finally, a significance test is performed to quantify the strength of evidence supporting a difference in isoform usage between the two subpopulations. SigFuge is available as an R package through Bioconductor.

In this paper, we first describe SigFuge using a simple toy example. We then compare the performance of our method against the closest competing approaches, DEXUS and SIBER, through an extensive simulation study. Finally, we apply our method to collections of lung squamous cell carcinoma (lung SQCC) and head and neck squamous cell carcinoma (head and neck SQCC) RNA-seq samples from the Cancer Genome Atlas (TCGA). We show that SigFuge identifies important transcriptional alterations including alternative splicing of the tumor suppressor gene *CDKN2A*. These results demonstrate that SigFuge is a powerful tool for identifying genes exhibiting differential isoform usage across large RNA-seq cohorts.

## MATERIALS AND METHODS

### SigFuge method

**Figure 1.**
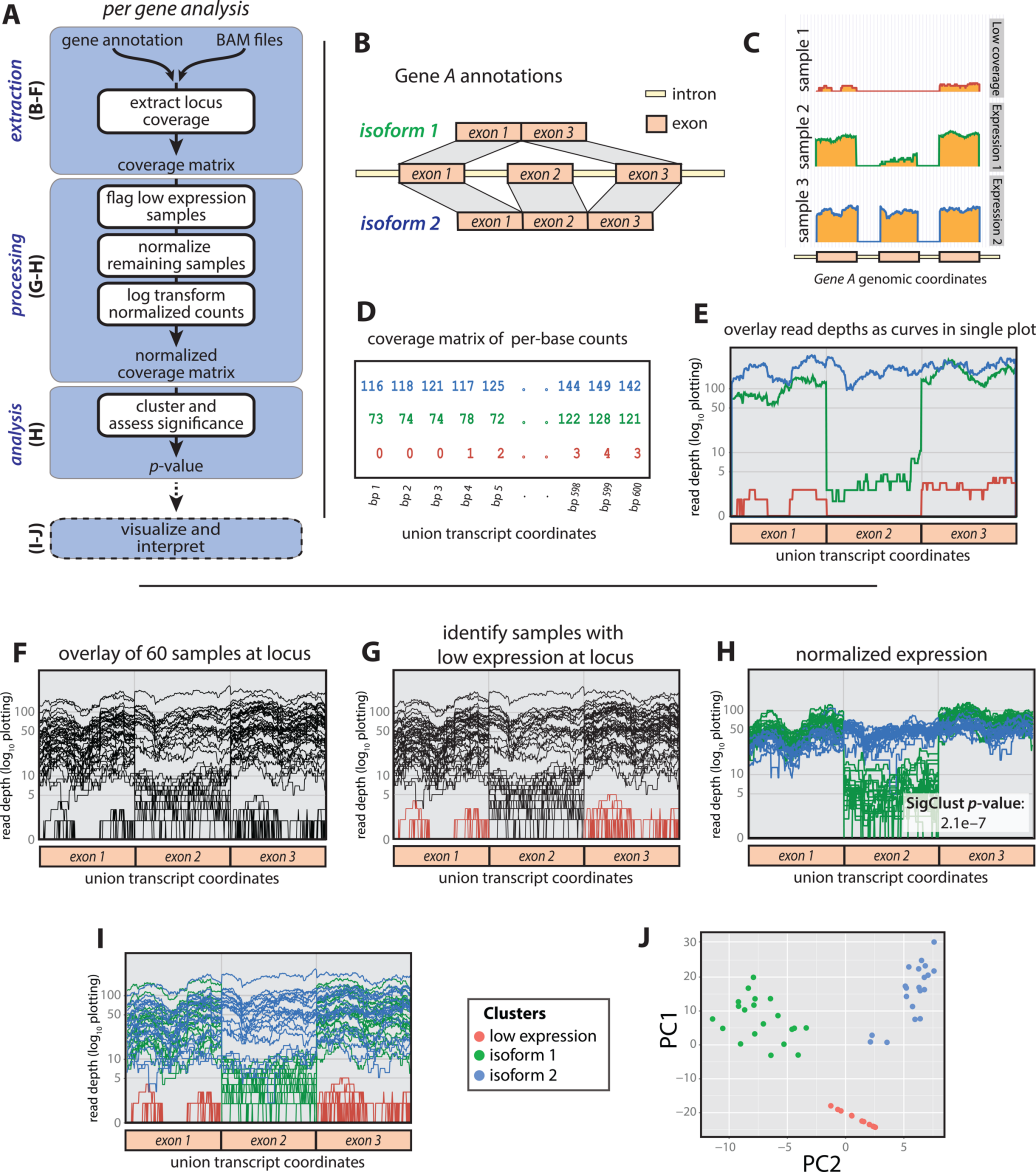
The SigFuge approach is illustrated through a hypothetical example *Gene A* with two true isoforms differing by a single cassette exon. (**A**) A general outline is given for the complete SigFuge pipeline. (**B**) The gene model includes two isoforms. (**C**) Read count pile-ups for samples 1, 2 and 3 show low expression (top, red), expression of isoform 1 (middle, green) and expression of isoform 2 (bottom, blue), respectively. (**D**) The expression curves are analyzed as a (sample × base) matrix representation of read counts. (**E**) The SigFuge approach studies these expression profiles as curves along the union gene model of the locus. (**F**) We consider a collection of 60 unlabeled samples at *Gene A*. (**G**) To study expression patterns, lowly expressed samples (colored red) are first excluded. (**H**) The remaining samples are count normalized, log-transformed and clustered using *K*-means clustering. The clusters are clearly visible in the log-transformed raw curve space (**I**) as well as the principal components space (**J**).

We describe the SigFuge method in three major parts: data extraction, processing and analysis. A pipeline of the complete approach is given in Figure 1A, with blue boxes used to distinguish the three parts. In the next subsections, we describe each part in detail, motivating our approach using a hypothetical *Gene A* across a cohort of 60 RNA-seq samples. To replicate the variation observed in RNA-seq data, per-base-position read counts were obtained from 60 of the lung SQCC samples along a subset of the bases within the *FAM64A* locus.

#### Data extraction

Consider *Gene A* having two known isoforms differing by a single cassette (middle) exon (Figure 1B). We first study three samples that represent important modes of expression in the larger cohort of 60 samples:

- low expression across the entire gene,
- primary expression of isoform 1,
- primary expression of isoform 2.

The differences in mRNA product are clearly reflected in the corresponding per-base read depths, plotted on the log-scale (Figure 1C). From this, we propose characterizing gene expression at the per-base resolution to study differential isoform usage. More specifically, we define a sample expression profile at a given gene to be the vector of per-base read depths across the exons of the union gene. In our toy example, the union gene, formed by combining all isoforms, is simply isoform 2 (Figure 1B). The resulting data structure is an expression count matrix with rows corresponding to samples, and columns corresponding to positions across the gene model (Figure 1D). This count matrix serves as the input to the computational steps of SigFuge, and can be obtained, for example, from BAM files using the SAMtools package (18). While read depth is commonly plotted using separate panels for each sample (Figure 1C), we prefer a more compact visualization where expression profiles are overlaid as curves in a single figure (Figure 1E). Note that the empty regions of Figure 1C corresponding to introns are excluded from the expression profiles of Figure 1E.

While exon annotations are not explicitly required for SigFuge, as each base-position is treated with equal weight, their use leads to more naturally interpretable results by restricting attention to expressed regions of the locus, e.g. in obtaining Figure 1E from Figure 1C. Similarly, our approach does not require information about the possible isoforms composing a gene model. This is a major strength of the method, as the precise structure of isoforms may be unknown.

#### Data processing

In Figure 1F–J, we consider the complete collection of 60 expression curves (samples) along *Gene A*. The goal of SigFuge is to determine whether the sample-set contains subgroups, or clusters, exhibiting different isoform usage. An example of differential isoform usage would be a subset of samples that only express isoform 1 while all remaining samples express both isoforms 1 and 2 in equal proportions. Since we are not interested in whole-gene changes in expression, we first perform a normalization of the sample curves to make identifying clusters of differential isoform usage easier. The normalization procedure is broken into the three following steps:

- Filtering: removing low-expression samples,
- Count normalizing: scaling expression curves to have equal total coverage,
- Log-transformation: mapping count data, which vary by orders of magnitude, to a more natural scale.

Since our interest is in differential isoform usage, samples not expressing any isoforms of the gene are first removed from the analysis, forming a separate cluster of low-expression samples. Specifically, at each locus we exclude samples with over 90% of base-positions having zero coverage, as well as samples with median coverage of less than five reads across covered positions. In the toy example, low-expression samples are colored red in Figure 1G and completely removed from Figure 1H. Next, count normalization is used to remove differences in overall gene expression between samples. To do this, each remaining sample is scaled by its total expression across the gene, i.e. by the corresponding row sum of the count matrix. While several approaches have been proposed for the normalization of RNA-seq expression data (19–21), these approaches were developed for genome-wide normalization, with the goal of identifying differentially expressed genes. In contrast, we aim to identify isoform imbalances at individual gene loci by identifying curves exhibiting different shapes, regardless of overall expression. Therefore, rather than employing normalization procedures for genome-wide differences (e.g. library sizes), we instead use a simple per-gene approach. Note that this normalization procedure assumes that each locus contains a single gene and may not be appropriate for loci containing multiple genes. Lastly, the scaled count data are log-transformed (Figure 1H). Log-transformation is used to study counts on the scale of relative expression, and is often applied when data vary over several orders of magnitude, as with read counts. To ensure the log is always well defined, i.e. to handle zero counts, all scaled values are increased by 1 prior to transformation. Note that zero counts remain at zero after transformation. As shown in Figure 1H, normalization reduces sample variability across most of the gene (exons 1 and 3), and highlights regions with non-uniform usage across samples (exon 2).

#### Data analysis

Following normalization, *K*-means clustering (22) for two clusters (*K* = 2) using Euclidean distance is applied to the normalized samples to identify clusters corresponding to differential isoform usage. In Figure 1H, these clusters, colored blue and green, differ noticeably by their use of exon 2. Here, SigFuge accurately captures clusters of samples with differing preferences for isoforms 1 and 2.

Translating the cluster labels to the original expression profiles, i.e. coloring the data by clusters, verifies that the identified clusters indeed correspond to clear differential patterns across *Gene A* (Figure 1I). To emphasize the notion of isoform clusters, we visualize the toy example using principal component analysis (23), an exploratory analysis tool for identifying low-dimensional structure in high-dimensional data (Figure 1J). The log-transformed raw data are projected along the first two principal component directions and colored according to the results of SigFuge. The plot clearly reveals three distinct clusters, showing the protocol accurately captures the main modes of variation among the samples.

This toy example was generated such that the clusters represent clearly differential patterns of expression. However, often loci considered in practice will only possess a single expression pattern. This may correspond to loci with a single dominant isoform or expression of multiple isoforms in similar proportions across all samples. As an exploratory tool, *K*-means identify clusters regardless of whether they represent true underlying structure. An important, yet difficult task in cluster analysis is to distinguish natural clustering from artificial clustering generated by the chosen algorithm. In the present context, this corresponds to distinguishing the small subset of genes with clusters exhibiting true differential isoform usage from the large number of genes across the entire transcriptome. To address this issue, SigFuge calculates a *p*-value quantifying the statistical significance of clustering at each locus. The SigFuge *p*-values can then be used to order a large set of genes to identify a subset of loci most likely to exhibit true differential isoform usage. An approach for determining the regions of differential usage is described in Supplementary Methods S3.

The *p*-value calculation is carried out using Statistical Significance of Clustering (SigClust) (24). Briefly, SigClust is a simulation-based hypothesis test proposed for analyzing clustering when the number of measurements greatly exceeds the number of observations. The procedure tests whether the two observed clusters are tighter than would be expected if the samples were drawn from a single Gaussian cluster. If the clusters are tighter, e.g. if the expression profiles are generated from two distinct patterns, we expect to observe a smaller *p*-value. For SigClust, the tightness of clusters is quantified using the 2-means cluster index (CI), a scaled version of the usual objective function for *K*-means clustering with two clusters. The behavior of the 2-means CI under the single Gaussian null is approximated using a large number of simulated null datasets. In our genome-wide analysis, we restrict the number of simulations to 100 for each gene. We then fit a Gaussian distribution to the 100 observed null 2-means CIs and report the lower tail probability of this fitted Gaussian as our approximate *p*-value, as described in (24). Although these *p*-values are not exact, they give a good sense of the relative significance of genes, which otherwise report equivalent empirical *p*-values of 0. While SigFuge may be used to test for the statistical significance of clusters obtained using any algorithm, *K*-means clustering is used as it has favorable properties for the SigClust testing procedure, as noted in (24). For the clustering shown in Figure 1H, SigFuge reports a significant *p*-value of 2.1 × 10^−7^. A more in-depth discussion of the SigClust assumptions as they pertain to our application can be found in Supplementary Methods S1 and Supplementary Figure S1. Finally, our analysis is restricted to *K* = 2 clusters as the SigClust methodology is currently only capable of testing for statistical significance with two subgroups. However, we note that other existing unsupervised approaches, such as SIBER and DEXUS, also seek a binary partition of observations, the latter separating between a single major condition and all remaining minor conditions.

When applying SigFuge to a large number of genes, we suggest using an appropriate statistical procedure for controlling either the family-wise error rate or false discovery rate (FDR). We use the Benjamini–Hochberg step-up procedure to control the FDR (25).

### Exon analysis

Commonly, RNA-seq-based expression data are obtained by aggregating and processing the per-base count data, which form the basis for our SigFuge approach. Measures such as RPKM (reads per kilobase per million mapped reads) (19) provide expression summaries of either the entire gene, or a portion of the gene, e.g. a transcript or an exon. These summaries are generally based on annotations obtained from public datasets such as RefSeq (26). As a preliminary assessment of any advantages to retaining the per-base resolution over more conventional summaries of the data, the SigFuge analysis was repeated using exon expression values, quantified by RPKM. In this analysis, for each gene, an exon expression matrix was passed to the processing and analysis steps of Figure 1A. The expression threshold was set such that samples having no exons with RPKM >1 were removed.

### Simulated RNA-seq study

An extensive simulation study was carried out at the level of single gene loci for varying experimental conditions. Datasets were simulated with several values of sample size, depth, dispersion and underlying isoform structure. For each simulation, per-base expression profiles were generated from an underlying gene model encoding two isoforms. Toy diagrams for the gene models used in the simulations are shown in Supplementary Figure S6. These include a three-exon gene model containing a cassette exon, as in Figure 1, and a four-exon gene model containing alternate cassette exons, such that two three-exon transcripts are encoded using distinct second exons. Expression profiles along a single gene locus were generated from two subpopulations differing only by their isoform preferences. That is, samples from the two subpopulations were simulated with the same expected gene-level expression, but with differing expected isoform-level expressions. The null setting with no differential behavior was also considered by setting the isoform preferences to be equal between the two subpopulations. A negative binomial distribution was used to generate isoform-level expression. Candidate values for the dispersion parameter, *ϕ*, were chosen as the quartiles of a lognormal distribution estimated in (27) for the Gilad dataset (28). A more detailed description of how datasets were generated is given in Supplementary Methods S2. Additionally, sample coverage plots for each of the five forms of differential isoform usage are given in Supplementary Figure S7. Simulations were repeated 100 times.

Each simulated single gene dataset was analyzed using SigFuge, DEXUS and SIBER. While DEXUS and SIBER were originally described for gene-level analysis, to make the approaches more comparable to SigFuge, both methods were applied to read counts at the following three levels of aggregation: (1) whole gene, (2) exon and (3) disjoint 100bp windows. DEXUS results were called significant according to the default informative/non-informative (I/NI) value threshold of the accompanying R implementation. Significance for the results of SIBER were determined based on a bimodality index (BI) cutoff described in Table 1 of (16) for controlling FDR at 0.05. Results for SIBER are not reported for total sample sizes <50, as BI cutoffs were only provided for sample sizes of 50, 100, 200 and 300. Since no clear approach exists for aggregating across multiple tests with the respective I/NI and BI output of DEXUS and SIBER, for the exon and 100bp window implementations of these two methods, loci were determined to be significant if any exon or window was called significant with no correction for multiple testing. SigFuge results were called significant at a *p*-value cutoff of 0.05.

**Table 1.** Results for select simulation settings, with parameters: *n*1, *n*2 (subpopulation sample sizes), *d* (gene length), *μ* (gene-level read depth) and *ϕ* (isoform-level dispersion). Parameter values *n*1, *n*2 = 50, 50; *d* = 1200; *μ* = 100; *φ* = 0.179 are treated as baseline, and deviations are marked by underlined values. For each setting, the numbers of significant calls out of 100 replications are reported for the default implementation of SigFuge, and DEXUS and SIBER at the gene, exon and 100bp levels of aggregation. The mean (standard deviation, SD) runtimes for single replications are reported in seconds for SigFuge, and DEXUS and SIBER at the 100bp level. Occasionally, NAs were reported in the output of SIBER. In this case, we mark the output with an asterisk (*m) and report the number of significant calls out of *m* < 100 simulations. For non-null simulations (settings 2, 3), the method with highest sensitivity is highlighted in bold. Complete simulation results may be found in Supplementary Table S3

**Simulation parameters**					**SigFuge**		**DEXUS**				**SIBER**			
**Setting**	*n*_1_, *n*_2_	*d*	$\boldsymbol{\mu }$	$\boldsymbol{\phi }$	**bp**	Runtime (SD)	**Gene**	**Exon**	**100bp**	Runtime (SD)	**Gene**	**Exon**	**100bp**	Runtime (SD)
1	100	1200	100	0.179	2	6.82 (0.31)	0	0	1	0.58 (0.01)	5	7	14	1.46 (0.26)
1	100	1200	500	0.369	7	6.59 (0.18)	0	2	2	0.58 (0.01)	2	5	9	1.55 (0.31)
1	20	1200	100	0.179	1	3.29 (0.14)	17	48	79	0.16 (0.01)	–	–	–	–
2	50, 50	1200	100	0.179	**89**	6.77 (0.23)	0	13	21	0.60 (0.01)	1	11	25	1.51 (0.26)
2	75, 25	1200	100	0.179	**98**	6.86 (0.24)	0	10	17	0.60 (0.03)	2	16	21	1.47 (0.26)
2	10, 10	1200	100	0.179	38	3.24 (0.12)	23	76	**86**	0.17 (0.00)	–	–	–	–
2	100, 100	1200	100	0.179	**98**	11.5 (0.21)	0	0	0	1.16 (0.01)	0	9	18	2.51 (0.40)
2	50, 50	1200	100	0.087	**100**	6.71 (0.18)	0	47	52	0.41 (0.02)	3	86	91	1.08 (0.14)
2	50, 50	1200	100	0.369	**62**	6.66 (0.18)	0	10	16	0.40 (0.01)	2	3	4*^99^	0.94 (0.22)
3	50, 50	1200	100	0.179	**99**	6.82 (0.18)	0	23	33	0.62 (0.11)	3	26	38	1.50 (0.17)
3	75, 25	1200	100	0.179	60	6.80 (0.19)	0	56	**72**	0.61 (0.01)	1	13	15	1.40 (0.19)
3	10, 10	1200	100	0.179	29	3.25 (0.16)	14	93	**96**	0.17 (0.00)	–	–	–	–

In addition to single gene simulations, a joint simulation of 10 000 genes was also considered, including 9000 null genes with no subpopulation behavior and 1000 non-null genes with varying levels of differential usage. Each simulated gene was again analyzed using SigFuge, as well as DEXUS and SIBER applied at three levels of aggregation. The SigFuge *p*-value, DEXUS I/NI and SIBER BI output were recorded for each simulated gene. As above, for the exon and 100bp window implementations of DEXUS and SIBER, the maximum I/NI and BI output were used to aggregate across the multiple tests. A more thorough description of the joint simulation setting is given in Supplementary Methods S2.

### TCGA lung squamous cell RNA-seq data

A collection of 177 lung SQCC RNA-seq samples was obtained from the TCGA Research Network. The dataset was processed as described in (29). The SAMtools depth function was used to obtain per-base read counts. Union gene models and corresponding composite exon boundaries for 20 500 genes were obtained from the TCGA generic annotation file v2.1 (https://tcga-data.nci.nih.gov/docs/GAF/GAF.hg19.June2011.bundle/outputs/) based on the December 2009 version of the UCSC Gene annotations. Methylation, mutation and copy number calls for the *CDKN2A* locus were also obtained from the supplementary data for (29).

### TCGA head and neck squamous cell RNA-seq data

A collection of 279 head and neck SQCC RNA-seq samples was obtained from the TCGA Research Network. The dataset was processed as described in (29). Per-base read counts were extracted for 20 500 genes as described above.

### Gel polymerase chain reaction validation

One novel event identified in the lung SQCC analysis, occurring at the *KLK12* locus, was selected for parallel structural validation. To validate our identified isoform clusters in *KLK12*, gel polymerase chain reaction (PCR) was carried out on two representative samples. RNA was reverse transcribed to cDNA using an iScript Reverse Transcription kit (Bio-Rad, Hercules, CA, USA) according to manufacturer's instructions. Amplification of the *KLK12* gene cDNA was performed using primers described previously (30). Briefly, 300ng of cDNA were amplified in a 50μL reaction containing 0.4μM of each primer, 0.2μM dNTPs, 1U of iProof High-Fidelity DNA Polymerase and its corresponding buffer (Bio-Rad, Hercules, CA, USA). Reactions were performed using a Bio-Rad C1000 thermocycler and the following cycling conditions: 98º C for 30 seconds, 35 repeats of 98 ºC for 30 seconds, 68 ºC for 50 seconds and 72 ºC for 1 minute, followed by a final extension at 72 ºC for 5 minutes. PCR products were resolved on a 2% agarose gel and results documented using a VersDoc gel imaging system (Bio-Rad, Hercules, CA, USA).

### Software availability

SigFuge is available in R as a package through Bioconductor (http://www.bioconductor.org/packages/devel/bioc/html/SigFuge.html). In addition to methods for clustering and calculating significance, the package includes functions for creating plots of per-base expression (expression plots) corresponding to our analysis approach. Figures 1E–I, 3 and 4 were constructed using this package.

## RESULTS

### Simulated RNA-seq analysis

To evaluate the performance of SigFuge under varying experimental conditions, simulation studies were carried out at the level of single gene loci and as a joint simulation of 10 000 loci. Results of representative simulations from the single gene study are presented in Table 1, with complete simulation results available in Supplementary Table S3. In Table 1, we report results for three simulation settings: (1) no differential behavior, (2) differential usage with a three-exon gene model and (3) differential usage with a four-exon gene model. The corresponding three and four-exon gene models are shown in Supplementary Figures S6 and S7. For each setting and combination of simulation parameters, i.e. row in Table 1, we report the number of significant calls over 100 replications of a single gene dataset. Here, the 100 replications serve to capture the sampling variation across replications of the experiment, and are not meant to represent a collection of 100 independent genes in a single experiment. To help orient the reader, consider the fourth row in Table 1. Under Simulation Setting 2, with subpopulation sample sizes 50 and 50, transcript length 1200, average depth 100 and dispersion 0.179, over 100 replications we observed 89 true positive calls by SigFuge, and 0, 13 and 21 true positive calls by DEXUS applied at the gene, exon and 100bp levels of aggregation. Similarly, we observed 1, 11 and 25 true positive calls by SIBER applied at the three levels of aggregation.

#### Setting 1

We first considered the null setting with no subpopulation differences. For all parameter values considered, SigFuge made only the expected number of false positive calls at the 0.05 significance level. Similarly, SIBER and DEXUS make few false positive calls for the larger sample size (*n* = 100). However, when the sample size was decreased (*n* = 20), DEXUS produced a large number of false significant calls across all levels of aggregation. With both DEXUS and SIBER, more false significant calls were observed at finer levels of aggregation, i.e. using exon and 100bp windows, as no correction was made for multiple testing at these levels.

#### Setting 2

This setting features differential usage across a three-exon gene model encoding for two isoforms, with samples drawn from subpopulations 1 and 2 expressing the isoforms at proportions 1:3 and 3:1, respectively. Notably, SigFuge consistently provided high sensitivity, with the exception of when sample size was decreased (*n*_1_, *n*_2_ = 10, 10). Furthermore, we observed expected trends across all methods, with sensitivity decreasing with increasing dispersion (*ϕ*), and increasing with greater sample size (*n*_1_, *n*_2_). In most settings other than low dispersion (*ϕ* = 0.087), DEXUS and SIBER showed low sensitivity across all levels of aggregation, with the exception of DEXUS showing high sensitivity with lower sample size. However, care is needed in interpreting this because of the poor specificity shown above for DEXUS in this context.

#### Setting 3

In this setting, we considered similar differential usage as in Setting 2, using a four-exon gene model. Samples were again drawn from two subpopulations expressing two isoforms at proportions 1:3 and 3:1. Similar results were observed as in Setting 2, with the exception of increased sensitivity by DEXUS and decreased sensitivity by SigFuge in the unbalanced sample size setting (*n*_1_, *n*_2_ = 75, 25). In general, sensitivity for both DEXUS and SIBER were higher in Setting 3, likely due to the regions of differential usage comprising a larger proportion of the entire gene.

#### Joint setting

**Figure 2.**
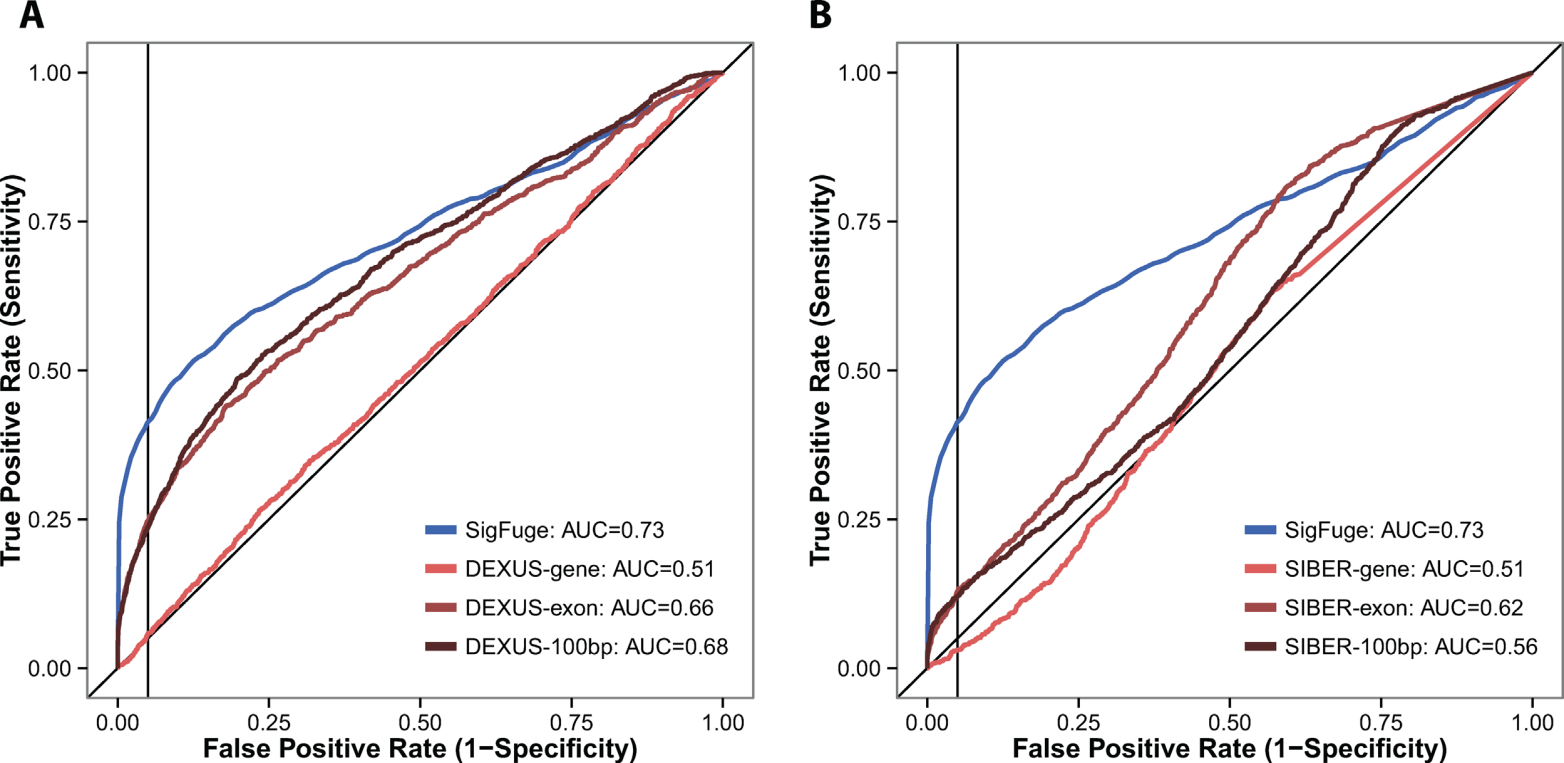
ROC curves are shown for SigFuge and six competing approaches applied to 10 000 simulated gene loci. Comparisons are given for SigFuge *p*-values and (**A**) DEXUS I/NI indices and (**B**) SIBER BI indices at the gene, exon and 100bp window levels. The corresponding AUC is reported for each method in the legends, and vertical black lines are used to denote the 95% specificity cutoff.

A joint simulation of 10 000 genes, including 9000 null and 1000 non-null genes was also performed to further evaluate the sensitivity and specificity of SigFuge, DEXUS and SIBER. The resulting receiver operating characteristic (ROC) curves for each method are given in Figure 2, and corresponding summary statistics are reported in Table 2, including area under the ROC curve (AUC), sensitivity and the F1-measure (the harmonic mean of precision and recall) (31). Across all metrics, SigFuge performs the best, with DEXUS consistently outperforming SIBER. Although the exon and 100bp window implementations of DEXUS achieve nearly the same AUC as SigFuge, the DEXUS-based approaches achieve substantially lower sensitivity when specificity is constrained to be above 90 or 95%.

**Table 2.** Summary statistics for the joint simulation, including AUC, sensitivity at 90 and 95% specificity (TPR_90_, TPR_95_), and the F1-measure at 90 and 95% specificity (F1_90_, F1_95_).

**Method**	**AUC**	**TPR_95_**	**TPR_90_**	**F1_95_**	**F1_90_**
SigFuge-bp	**0.73**	**0.41**	**0.49**	**0.44**	**0.41**
DEXUS-gene	0.51	0.06	0.11	0.08	0.11
DEXUS-exon	0.66	0.25	0.34	0.29	0.30
DEXUS-100bp	0.68	0.24	0.34	0.28	0.30
SIBER-gene	0.51	0.03	0.06	0.04	0.06
SIBER-exon	0.62	0.13	0.17	0.16	0.17
SIBER-100bp	0.56	0.12	0.17	0.15	0.16

In general, SigFuge was found to produce higher sensitivity and lower false positive calls than either DEXUS or SIBER. Additionally, in both Settings 2 and 3, DEXUS and SIBER benefited substantially from aggregating at the exon and 100bp window level. Finally, we note that for all settings, SigFuge required the most computational time, with DEXUS requiring the least. This is a consequence of the simulation-based *p*-value calculation implemented by SigFuge and the underlying SigClust algorithm. However, as computational times differ with available hardware, it may be more appropriate to interpret these results as a relative, rather than absolute, comparison of computational cost across the evaluated methods. Furthermore, since SigFuge is applied at each locus separately, the method may be easily parallelized on a cluster to reduce the total computing time for larger scale analyses.

### Lung SQCC analysis

To illustrate the power of our approach in real data, we applied SigFuge to a set of 177 lung SQCC samples. Of the 20 500 genes considered, 3547 genes having <10 samples passing the expression threshold were removed from the analysis. Genes of this type were empirically considered to be expressed at such low levels in so few samples that clustering results would be of little interest. The distribution of the remaining 16 953 *p*-values is shown in Supplementary Figure S2.

Controlling FDR at 5%, 322 genes were identified as showing significant differential patterns of expression. Manual review of the expression at these genes suggested that SigFuge identified a limited number of recurring patterns. Thus, the set of 322 genes was separated into six categories by visually inspecting the corresponding expression plot at each locus (Table 3). Genes placed in the same category were determined to exhibit similar patterns of differential isoform usage. While these categories do not necessarily correspond to unique regulatory events, they help summarize the various types of differences detectable by SigFuge. A complete list of the top 322 genes and their corresponding categories and SigFuge *p*-values may be found in Supplementary Table S1.

**Table 3.** Six consistent patterns of differential usage were identified.

**Cat.**	**Name**	**Count**	**Representative genes**
1	Cassette exon	27	*CDKN2A*, *KLK12*, *FAM64A*
2	Outliers	67	*APRT*, *RABAC1*, *TSPO*
3	Differential use of 5′- exons	50	*SPATA21*, *SMN1*, *CKMT1A*
4	Differential use of 3′- exons	15	*RPL22L1*, *CRHR1*, *ECE2*
5	Alternative start sites	53	*RPS8*, *RPL7A*, *RPL35A*
6	Likely mapping artifacts	110	*S100A7*, *HLA-DRB1*, *RPL27*

The first five categories, containing potentially biologically meaningful behavior, include: (1) skipping of a cassette exon, (2) outlier behavior, i.e. differential usage in less than five samples, (3) differential use of the 5′- end, (iv) differential use of the 3′-end and (v) alternative start sites. Further details on how genes were divided among the six categories may be found in Supplementary Results S1.

**Figure 3.**
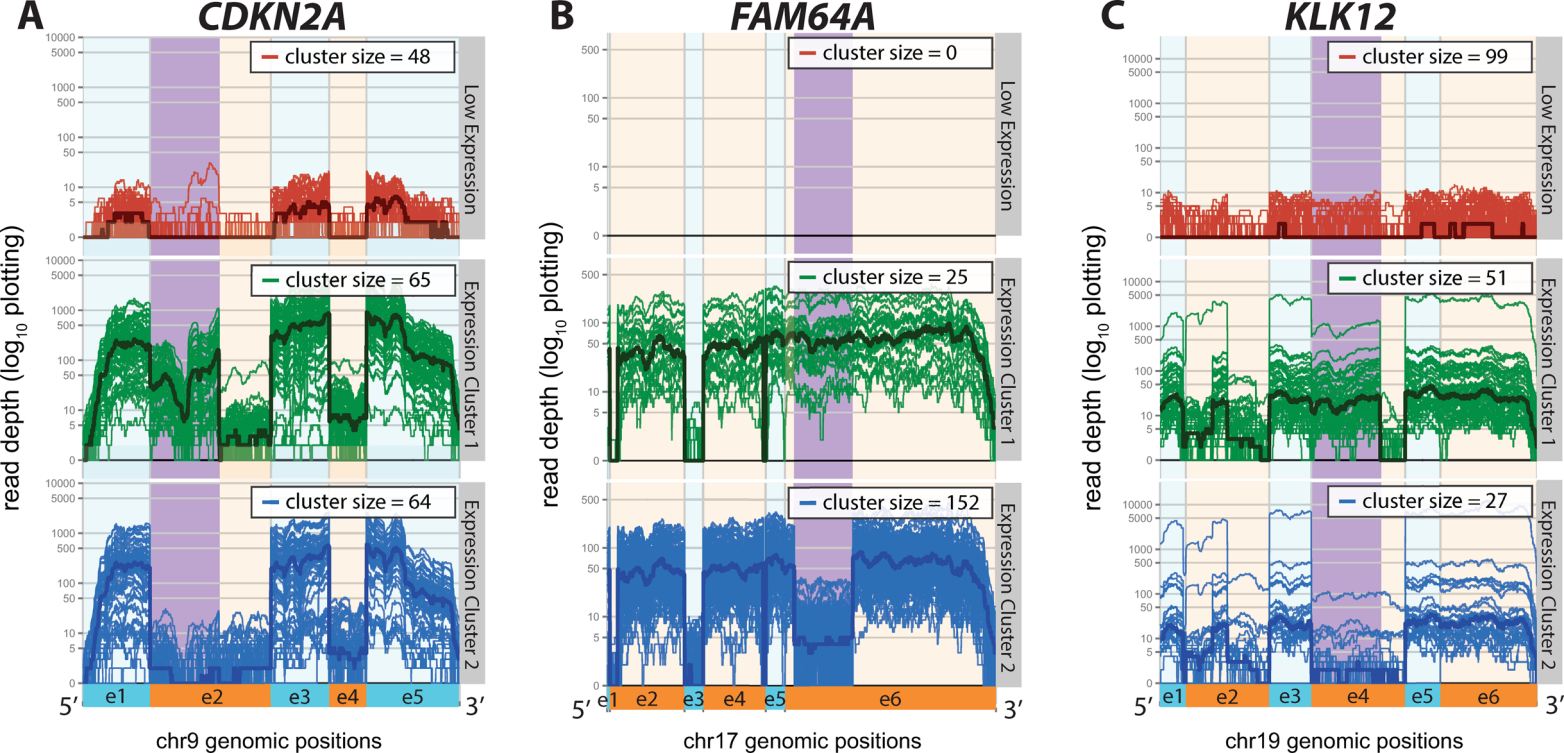
Three genes identified as being significant by SigFuge with differential usage of middle exons (Cat. 1) are shown: (**A**) *CDKN2A*, (**B**) *FAM64A*, and (**C**) *KLK12*. For each gene, the SigFuge clusters are shown using separate colors and panels. Each red, green and blue curve represents an individual sample and darker bold curves are used to denote the cluster medians. Regions of differential usage, identified by visual inspection, are highlighted in the figure for each gene. Alternating orange and blue are used to denote annotated exon boundaries.

**Figure 4.**
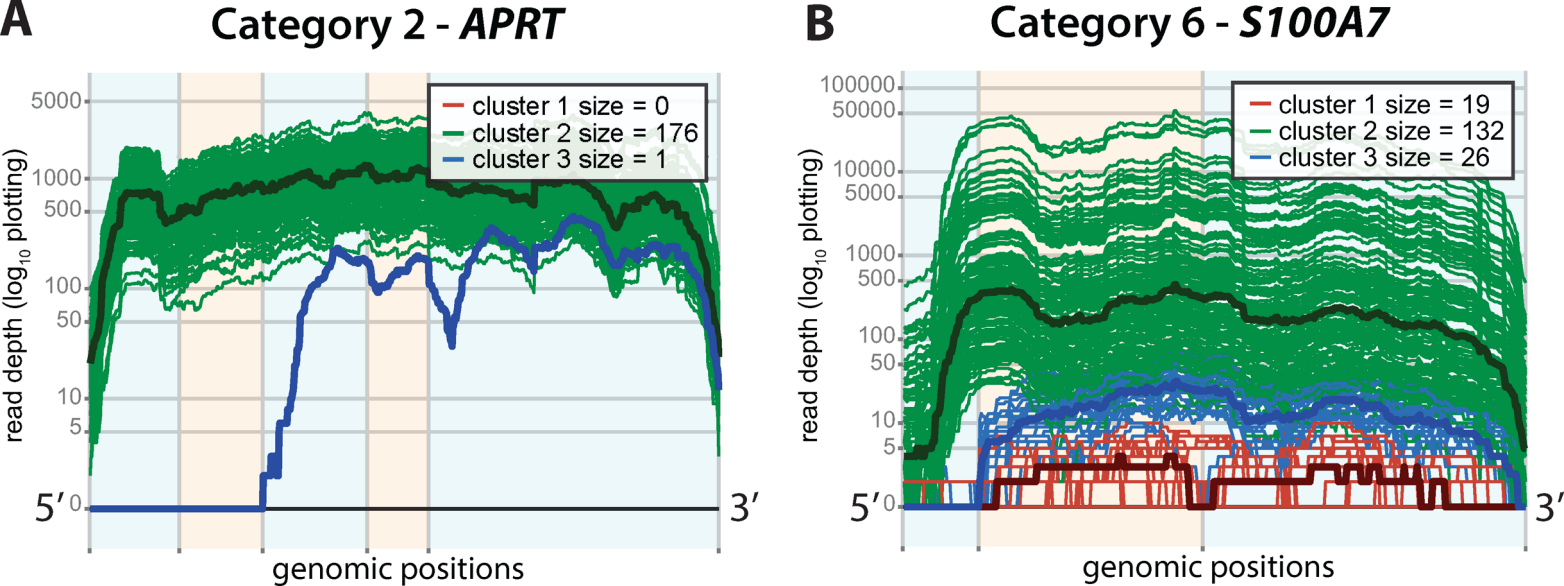
Representative genes for two common patterns of differential expression are shown: (**A**) a single outlier sample (Cat. 2), *APRT* and (**B**) an unmapped short starting exon (Cat. 6), *S100A7*. Alternating orange and blue are used to denote annotated exon boundaries. Red, blue and green represent clusters of low expression, isoform usage 1 and isoform usage 2. Bold lines denote cluster median expression.

In Figure 3, we show the expression plots for three genes with differential usage of a cassette exon (Cat. 1), each described in detail later in this section. In each plot, the region of differential expression along the transcript is highlighted in purple. Additionally, in Figure 4A, we show *APRT*, an example of a gene with one clear outlier sample (Cat. 2), and provide examples of genes from Categories 3–5 in Supplementary Figure S3. We primarily focus on the set of genes in Category 1, as their functional impact is most directly predicted.

Events deemed likely to be artifacts of current RNA-seq technologies and alignment algorithms, such as short unmapped exons (*S100A7*, Figure 4B), are included in Category 6. Previous studies have shown that many split-read alignment algorithms have difficulty aligning reads to short exons, especially when overall gene expression is low (32). Thus, it is highly likely that the identified clusters at *S100A7* simply correspond to samples falling above and below the threshold for properly aligning reads to the first exon.

The set of Category 1 genes includes 27 loci identified based on apparent gain or loss of a middle exon. We will now describe in detail three notable genes from this category for which differential isoform usage may play a role in tumor development and growth: *CDKN2A*, *FAM64A* and *KLK12*.

#### *CDKN2A* locus

*CDKN2A* is a tumor suppressor gene known to code for two proteins, $\text{p16}^{\text{INK4a}}$ and $\text{p14}^{\text{ARF}}$. Recently, *CDKN2A* was identified as one of the most highly altered genes in lung SQCC (29). In the union gene model shown in Figure 3A, expression of exons 1−3−5 encodes for $\text{p14}^{\text{ARF}}$, and expression of exons 2−3−5 encodes for $\text{p16}^{\text{INK4a}}$. Thus, the SigFuge classes correspond to expression of neither protein due to low expression (red class), expression of $\text{p16}^{\text{INK4a}}$ and $\text{p14}^{\text{ARF}}$ (green class) and expression of $\text{p14}^{\text{ARF}}$ only (blue class).

Table 4 compares the SigFuge clusters against the three major modes of *CDKN2A* inactivation identified by the TCGA integrative analysis: homozygous deletion, epigenetic silencing by methylation and inactivation by point mutation. As can be seen by the clear diagonal structure of the table, the SigFuge clusters approximately capture the three classes of alterations. Notably, 64% of samples in the blue class were methylated, including all but one methylated sample in the cohort. Furthermore, 96% of samples identified as low expression (red class) were homozygous deleted, comprising 82% of homozygous deleted samples in all clusters, confirming the validity of our proposed filtering scheme. Pearson's chi-square ($\chi$^2^) tests were applied to each row of Table 4 and the entire table. The highly significant *p*-values further confirm the strong association between our SigFuge clusters and the previously identified alterations.

**Table 4.** *CDKN2A* agreement between SigFuge labels and genomic alteration status.

	**SigFuge label**				
	**Red**	**Green**	**Blue**	**Total**	$\chi$2 *p*-value
**Homozygous deleted**	46	0	8	54	3 × 10^−15^
**Mutated**	0	29	3	32	4 × 10^−11^
**Methylated**	1	0	41	42	≈ 0
**None**	1	36	12	49	3 × 10^−9^
**Total**	48	65	64	177	≈ 0

#### *FAM64A* locus

*FAM64A* is a gene that has been implicated in the regulation of cell proliferation, suggesting a possible role in cancer (33). Further, *FAM64A* has been shown to be highly expressed in leukemia, lymphoma and other tumor cell lines (34). The plot of *FAM64A* expression shows clustering based on an unannotated splice junction, resulting in lower expression for a large proportion of the final exon (Figure 3B). Although the event has been previously reported as a retained intron (35), the implication of the isoform difference has yet to be described. This supports our use of per-base expression, as analysis based on aggregation along exon or whole gene boundaries would have likely missed this event.

#### *KLK12* locus

*KLK12* is part of a family of 15 kallikrein-related peptidases (*KLK* genes) encoding secreted serine proteases. *KLK* splice variants are receiving increased attention as potential biomarkers in cancer, and have been studied in epithelial ovarian, prostate and lung cancers (36–38).

The *KLK12* locus is known to produce multiple isoforms, largely differing by the use of a cassette exon, exon 4 (Figure 3C, exon 4). A recent study has shown expression of the exon 4 skipping isoform to be clinically relevant in breast cancer (30). The identified *KLK12* expression clusters capture evidence of similar differential usage in our cohort of 177 samples. These results support the potential of *KLK12* and other *KLK* splice variants as markers in lung SQCC.

To confirm our identified clusters were not an artifact of sequencing, we performed gel PCR at the *KLK12* gene locus on representative samples from each of the green and blue classes in Figure 3C. Our results confirm the absence of isoforms retaining exon 4 in the sample from the blue class (Supplementary Figure S4).

### Exon analysis

To examine whether the same transcriptional alterations could be identified using exon-level rather than base-level expression values, we performed a similar clustering analysis using exon RPKM values at each gene locus. Of the 20 500 genes considered, 4243 were excluded from the analysis for having <10 samples passing the expression threshold. Of the remaining 16 257 genes, 1898 genes were considered significant by a FDR cutoff of 5%. The distribution of all 16 257 *p*-values is shown in Supplementary Figure S5, and a list of the top 1898 genes identified by exon-level clustering, and their corresponding *p*-values may be found in Supplementary Table S2.

Of the 322 genes significant by our per-base SigFuge analysis, only 27 were identified as significant in the exon-level analysis. Of these, only five genes were from Category 1. The three genes considered in Figure 3, *CDKN2A*, *FAM64A* and *KLK12* had respective SigClust-like *p*-values: 0.011, 1.05*e* −28 and 0.9225, with only *FAM64A* considered significant by the FDR threshold. However, the significant clusters identified at *FAM64A* by exon-level expression completely miss the differential usage at exon 6 shown in Figure 3B. In fact, the clusters identified by the exon-level analysis were inconsistent with those shown in Figure 3 for all three genes (Supplementary Figure S5).

Here, we note that the exon-based analysis is simply a modification of the SigFuge approach, and may be considered an additional contribution of our work. Thus, while not investigated further, loci significant by the exon-level analysis may also reveal interesting transcriptional behavior, further validating our approach to clustering by alternate transcription patterns at single genes.

### Head and neck SQCC analysis

As an attempt to confirm the results identified in lung SQCC, SigFuge was also applied to an independent set of 279 head and neck SQCC samples, a biologically similar tumor type. Controlling FDR at 5%, 335 genes were identified as exhibiting significant differential usage. Notably, we identified similar clusters of differential isoform usage at the *CDKN2A*, *KLK12* and *FAM64A* loci shown in Figure 3 (Supplementary Figure S8). Of the three, *KLK12* and *FAM64A* were included in the set of 335 significant head and neck SQCC genes with *p*-values 1.99*e*−15, and 6.76*e*−6. While not included in the top 335 genes, *CDKN2A* was also found to exhibit strong evidence of differential isoform usage (*p*-value 0.0021, 381st most significant). Further, of the 27 Category 1 genes identified in lung SQCC, 21 (78%) were also identified as significant in head and neck SQCC, suggesting the reproducibility of most interesting events across different datasets.

## DISCUSSION

The introduction of RNA-seq has fundamentally transformed genomic research in cancer by making it possible to study transcriptomes at the resolution of base positions. Concurrently, the importance of studying isoform regulatory behavior beyond whole gene events has become increasingly clear. SigFuge is presented as a novel method capable of unsupervised discovery of differential isoform events in RNA-seq. Our approach to studying gene expression as per-base expression curves along transcriptome coordinates makes it possible to identify differential events without strictly constraining our analysis to proposed exon or transcript boundaries. However, we also note that as shown in our analysis, the approach may be easily extended to cluster based on exon-level expression, if desired. Furthermore, SigFuge could potentially be extended for the analysis of exon arrays in addition to RNA-seq, although more testing would be necessary to determine whether our normalization procedure would require any modification. Our implementation of SigFuge as an R package through Bioconductor makes the approach easily accessible for many investigators.

Through simulation study, we have shown that SigFuge is often capable of detecting true differential isoform usage with higher sensitivity than DEXUS and SIBER across various experimental conditions. This may be attributed to the unique multivariate approach taken by SigFuge, in which all base positions and exons are considered simultaneously to detect and assess clustering. In contrast, DEXUS and SIBER cluster marginally at individual genes or exons, thus rendering the approaches less sensitive to isoform differences which occur non-uniformly, but in concert, across the entire gene. In addition to having high sensitivity, we have shown that in the absence of subpopulation differences, SigFuge does not make more than the expected number of false positive calls.

Applying SigFuge to a cohort of lung SQCC samples, we identified *CDKN2A*, a tumor suppressor gene known to be highly altered in lung SQCC, and *KLK12*, a gene recently shown to have differential isoform usage in breast cancer. To our knowledge, SigFuge is the only unsupervised approach to identifying loci with significant differential isoform usage. All other genome-wide methods for identifying genes with differential isoform usage require *a priori* knowledge of the differential class labels, and therefore could not be used to identify these events. The biological relevance of our *CDKN2A* clusters was validated by observed high concordance with homozygous deletion, methylation and point mutation events at the locus. Further, the predicted isoforms of *KLK12* were confirmed by PCR as a validation of the method. Additionally, many of the clusters identified in lung SQCC were found to reproduce in an independent analysis of 279 head and neck SQCC samples, suggesting that our discoveries relate to biologically meaningful events.

The importance of alternative splicing in the development of diseases, including cancer, is well recognized (39, 40). SigFuge shows promise as a tool for identifying biologically relevant cases of aberrant isoform usage. Given clinical outcomes, testing clusters of differential isoform usage for significant associations with survival could potentially reveal novel therapeutic targets.

A major benefit of SigFuge is the calculation of a *p*-value to quantify significance of clustering. Using the *p*-value, it becomes possible to screen a large set of genes to identify a small subset of potentially biologically interesting loci. However, our post-hoc analysis makes it clear that identifying truly interesting events is not simply a statistical endeavor, i.e. finding significant SigFuge *p*-values. That is, some loci identified by SigFuge as statistically significant, may on manual review appear to be artifacts introduced by sequencing and mapping challenges beyond our control. Therefore, manual review of statistically significant results is strongly recommended. To this end, our approach to visualizing expression profiles makes it possible to quickly gain intuition at each locus to determine the nature of the underlying event.

In our analysis, to allow for a quick and interpretable evaluation of the results, SigFuge was implemented only in regions defined by annotated genes. However, gene annotations may be incomplete or inexact, reducing our power to detect novel isoform and splicing differences. A desirable modification of the current approach would be to use a guided gene discovery tool such as Cufflinks (41) or Scripture (42) to supplement the current annotation with additional candidate exons. Such an extension would allow a more unbiased exploration of the transcriptome and may lead to the discovery of additional alternative isoform usage events with SigFuge.

We conclude by introducing several proposed extensions of the current SigFuge approach. First, as seen in our application to the lung SQCC dataset (Figure 4 and Table 4), SigFuge is capable of identifying outlier events with few samples showing significantly altered transcriptional patterns. These outlier events may be attributable to unique fusion events or single nucleotide polymorphisms (SNPs) resulting in alteration of the start or end site of a gene. In some cases, these events may correspond to the loss of a tumor suppressor gene. While additional modifications would be needed to increase the specificity of SigFuge to these events, this presents an interesting direction of further development.

A limitation of the current SigFuge approach and underlying SigClust algorithm is the restriction to testing with only *K* = 2 clusters. An extension of SigClust to handle greater than two clusters could provide substantial benefit for SigFuge as well as more general applications. We are actively pursuing such an extension in related but separate work.

Finally, we propose extending SigFuge to more directly incorporate splicing information. In addition to estimating read depth at a higher resolution, RNA-seq also provides direct evidence for splicing through junction-spanning reads. Recently, methods such as SplicingCompass (43) have proposed using junction coverage to study differential splicing behavior. While the SigFuge approach only makes use of per-base coverage, it is not difficult to imagine combining the two sources of information to obtain a more complete view of isoform usage. As the number of junctions and the distribution of their coverage differ substantially from that of per-base expression, further investigation is necessary before moving forward. However, we believe that this direction shows great potential, and is actively advancing this work.

Recent genomics studies using RNA-seq are beginning to shed light on the sheer prevalence and importance of post-transcriptional events across the human genome. In this paper, we have proposed the first approach for the unsupervised discovery of differential isoform usage in RNA-seq data. By taking the novel approach of clustering by per-base expression, we believe SigFuge is a step in the right direction for realizing the full potential of RNA-seq for understanding the genomic complexity of diseases.

## SUPPLEMENTARY DATA

Supplementary Data are available at NAR Online.

SUPPLEMENTARY DATA
