# Supplementary material for: SigFuge: single gene clustering of RNA-seq reveals differential isoform usage among cancer samples
Source: Nucleic Acids Res. 2014 Jul 16;42(14):e113. doi: 10.1093/nar/gku521 (PMC4132703; doi:10.1093/nar/gku521)
Supplement: SUPPLEMENTARY DATA [file supp_42_14_e113__index.html]

SigFuge: single gene clustering of RNA-seq reveals differential isoform usage among cancer samples — SigFuge: single gene clustering of RNA-seq reveals differential isoform usage among cancer samples — SUPPLEMENTARY DATA 

# SigFuge: single gene clustering of RNA-seq reveals differential isoform usage among cancer samples

## SUPPLEMENTARY DATA

**Files in this Data Supplement:**

- SUPPLEMENTARY DATA
