# Supplementary material for: SigFuge: single gene clustering of RNA-seq reveals differential isoform usage among cancer samples
Source: Nucleic Acids Res. 2014 Jul 16;42(14):e113. doi: 10.1093/nar/gku521 (PMC4132703; doi:10.1093/nar/gku521)
Supplement: SUPPLEMENTARY DATA [file supp_gku521_nar-03595-met-z-2013-File012.zip › SigFuge-supplement/SigFuge_supp_R2.docx]

**Supplementary Materials – SigFuge: single gene clustering of RNA-seq reveals differential isoform usage among cancer samples.**

**Supplementary Methods S1 – SigClust validity and implementation**

**Supplementary Methods S2 – RNA-seq simulation study**

**Supplementary Methods S3 – Differential usage detection**

**Supplementary Figure S1 – PCA scatterplot for null gene**

**Supplementary Figure S2 – Lung SQCC results**

**Supplementary Figure S3 – Examples of genes from Categories 3-5**

**Supplementary Figure S4 – PCR results**

**Supplementary Figure S5 – Exon analysis results**

**Supplementary Figure S6 – RNA-seq simulation gene models**

**Supplementary Figure S7 – RNA-seq simulation settings**

**Supplementary Figure S8 – Head and neck SQCC clustering at *CDKN2A*, *FAM64A, KLK12***

**Supplementary Results S1 – Lung SQCC gene categorization**

**Supplementary Table S1 – Significant genes by SigFuge analysis**

**Supplementary Table S2 – Significant genes by exon-based analysis**

**Supplementary Table S3 – Additional RNA-seq simulation results**

**Supplementary Methods S1**

**SigClust validity and implementation**

In this section, we describe our choice of parameters in implementing SigClust for screening the set of lung SQCC data first. We then discuss the validity of the SigClust null Gaussian assumption in our application to normalized per-base expression.

**SigClust parameters**

We implemented SigClust using the sample covariance matrix to estimate the null Gaussian distribution. Extensive simulation study has shown that among several proposed SigClust covariance estimators, the sample covariance approach produces consistently conservative p-values (preprint, arXiv:1305.5879). For each gene, SigClust p-values were calculated using 100 simulated datasets. For each gene, a Gaussian-fit p-value was reported. These approximate p-values were calculated by the lower tail of the best fit Gaussian to the 100 cluster indices calculated from the simulated null data.

**SigClust Gaussian null assumption**

The SigClust null hypothesis states that the data are generated from a single Gaussian distribution with at most only a few directions of high variance, corresponding to signal, and the remaining directions spanned by a relatively low level of background noise. To test the validity of the null, we studied the expression of 33 genes with non-significant SigFuge p-values. Specifically, after ranking the 16,953 genes by their SigFuge p-value, we selected the genes with the 500th, 1,000th, …, 16,500th most significant p-values. For each of these genes, we examined scatterplots for the principal component scores along the first four principal component (PC) directions, and the corresponding screeplot, i.e. eigenvalues, of the processed coverage matrix passed to SigClust. An example for a single gene, ASRGL1, is shown in Figure S1. We show the 2-way scatterplots along the first four PC directions. The one-dimensional distribution of scores along each direction is shown in the diagonal plots. Each off-diagonal plot shows the data appear to be roughly spherical, suggesting the multivariate Gaussian assumption may be reasonable.

**Supplementary Methods S2**

**RNA-seq simulation study**

In this section, we describe how datasets of single gene expression profiles were simulated to analyze the performance of our SigFuge approach across various experimental settings. For each simulation setting, we generated expression profiles from two underlying subpopulations that differed only by their respective isoform preferences. That is, we simulated the dataset such that all samples had equivalent expected gene-level expression and isoform-level dispersion. Note that we also considered the null case with no subpopulation behavior by setting the isoform preferences to be equivalent between the two groups. Furthermore, note that the underlying subpopulation labels were only used in simulating the data and not during the later analysis steps, as our goal was to compare and evaluate the performance of unsupervised methods. Each simulation setting was repeated 100 times.

In each simulation, single gene datasets were simulated for loci encoding precisely two isoforms. Two gene models were considered: a three-exon cassette model, and a four-exon alternate cassette model (Supplementary Figure S6). Since each sample is quantified as a vector of per-base expression counts across exonic coordinates, it can be shown that the above two models are roughly equivalent, up to a re-ordering of the base positions, to a much larger class of differential isoform usage events. Throughout these simulations, each exon is of equal length.

For each of the two gene models, simulations were performed with the following set of experimental parameters, with corresponding values for simulation given in parentheses:

- **μ**: mean read depth (50; 100; 500)
- **φ**: dispersion (0.087; 0.179; 0.369)
- **d**: gene (exonic) length (1200; 2400)
- **n_1_**,**n_2_**: subgroup sample sizes (10,10; 50,50; 50,1; 75,25; 100,100)
- **ψ_1_**/**ψ_2_** = (ψ**_11_**, ψ**_12_**)/(ψ**_21_**, ψ**_22_**): subgroup isoform proportions such that ψ**_11_**+ψ**_12_** = ψ**_21_**+ψ**_22_** = 1 (see Supplementary Table S3).

For a fixed gene model (e.g. Supplementary Figure S6 panel A), **μ**, **φ**, **d**, **n_1_**,**n_2_**, **ψ_1_**/**ψ_2_**, a single gene dataset was simulated as follows. First, for the **n_1_** samples of subpopulation 1, isoform level expression values were simulated from two Negative Binomial distributions with means **μ***ψ**_11_** and **μ***ψ**_12_**, and dispersion parameter **φ**. Similarly, isoform level expression values were simulated for the **n**_2_ samples of subpopulation 2 with means **μ***ψ**_21_** and **μ***ψ**_22_**. Then, for each sample and each isoform, 50bp “reads” were generated randomly (uniformly) across the respective isoform model to achieve the necessary isoform level expression. That is, for example, to achieve isoform expression of **100** across a transcript of length **d**, **100*d**/**50** reads would be randomly generated across the transcript. Finally, at each position along the gene, the number of aligned reads was counted to produce the per-base level expression profiles passed to SigFuge. Examples of the resulting per-base profiles are shown in Supplementary Figure S7.

For input to DEXUS and SIBER, read counts were summarized within gene, exon and 100bp boundaries. The 100bp windows were generated by taking non-overlapping regions of the gene. For example, with a simulated 3-exon gene locus of length 1200bp, we perform 1 test at the gene level, 3 tests at the exon level, and 12 tests at the 100bp window levels. Windows of 100bp were chosen as a compromise between obtaining finer resolution than exons, and keeping the number of tests manageable, e.g. using 1bp windows would require summarizing the results of thousands of tests for each gene locus. DEXUS and SIBER were applied to the raw read counts within each bin with no normalization. Aside from specifying a Negative Binomial model for SIBER, the methods were applied using the default settings of their respective R implementations.

We consider the following 5 simulation settings (Supplementary Figure S7):

|  | Gene model | ψ**_11_** / ψ**_12_** | ψ**_21_** / ψ**_22_** |
| --- | --- | --- | --- |
| 1 | Three-exon | 0.50 / 0.50 | 0.50 / 0.50 |
| 2 | Three-exon | 0.25 / 0.75 | 0.75 / 0.25 |
| 3 | Four-exon | 0.25 / 0.75 | 0.75 / 0.25 |
| 4 | Three-exon | 0.67 / 0.33 | 0.33 / 0.67 |
| 5 | Four-exon | 0.67 / 0.33 | 0.33 / 0.67 |

For the joint simulation, 9,000 null and 1,000 non-null loci were simulated independently for 100 samples. Of the 9,000 null genes, 4,500 were simulated according to Setting 1, with the remaining 4,500 as in Setting 1, except with a four-exon gene model. The 1,000 non-null genes consisted of genes simulated according to Settings 2, 3, 4 and 5. 500 were simulated from either Settings 2 or 4 with equal probability, and similarly, the remaining 500 were simulated from either Settings 3 or 5. The remaining simulation parameters described above, **μ**, **φ**, **d**, **n_1_**,**n_2_**, were randomly selected from the following set of parameters, with equal probability. Note that in all cases **n_1_**+**n_2_**=100 such that the total sample size is always 100.

- **μ**: 50; 100; 200
- **φ**: 0.087; 0.179; 0.369
- **d**: 1200; 2400
- **n_1_**,**n_2_**: 10,90; 75,27; 50,50

**Supplementary Methods S3**

**Differential usage detection**

First, assume we have applied the SigFuge approach to identify two clusters with statistically significant differential isoform usage. Then, given the two cluster labels, we apply a classification algorithm, such as the support vector machine (SVM), to the normalized dataset, e.g. the data shown in Figure 1H. Here, note that the loadings of the resulting classifier specify a direction optimally separating the two classes according to some objective. We therefore use the loadings of the classifier to infer the regions of the gene-locus that most clearly distinguish the two clusters. That is, if expression along a set of base positions can be used to clearly separate the two clusters, we identify these base positions as corresponding to regions of differential usage.

**Supplementary Figure S1**

Figure S1: Checking SigClust distributional assumptions on ASRGL1, a gene with non-significant SigFuge p-value. Two-way scatterplots of PC scores along first 4 PC directions. Panels along the diagonal show the one-dimensional distribution of the set of scores along each PC direction.

**Supplementary Figure S2**

**Lung SQCC analysis results**

**
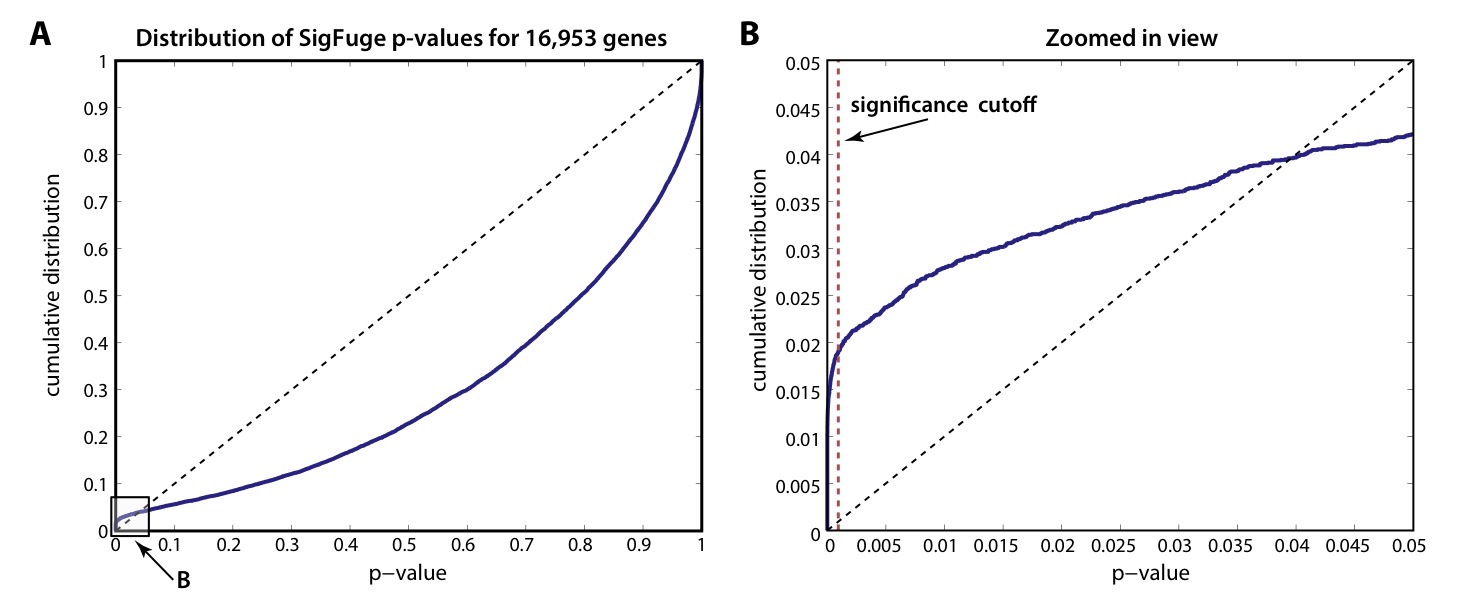
**Figure S2: Distribution of SigClust p-values for 16,953 genes with more than 10 highly expressed samples. A dashed red line is used to denote the p-value significance cutoff for controlling FDR at 5%. (A) Empirical cumulative distribution function (CDF) of p-values and (B) zoomed in view of the empirical CDF for the range of p-values < 0.05. The distribution of p-values is non-uniform with a large number of p-values near 1. This is likely due to our choice of SigClust parameters, which lead to conservative p-values, as described in Supplementary Methods S1.

**Supplementary Figure S3**

**Examples of genes form Categories 3-5**


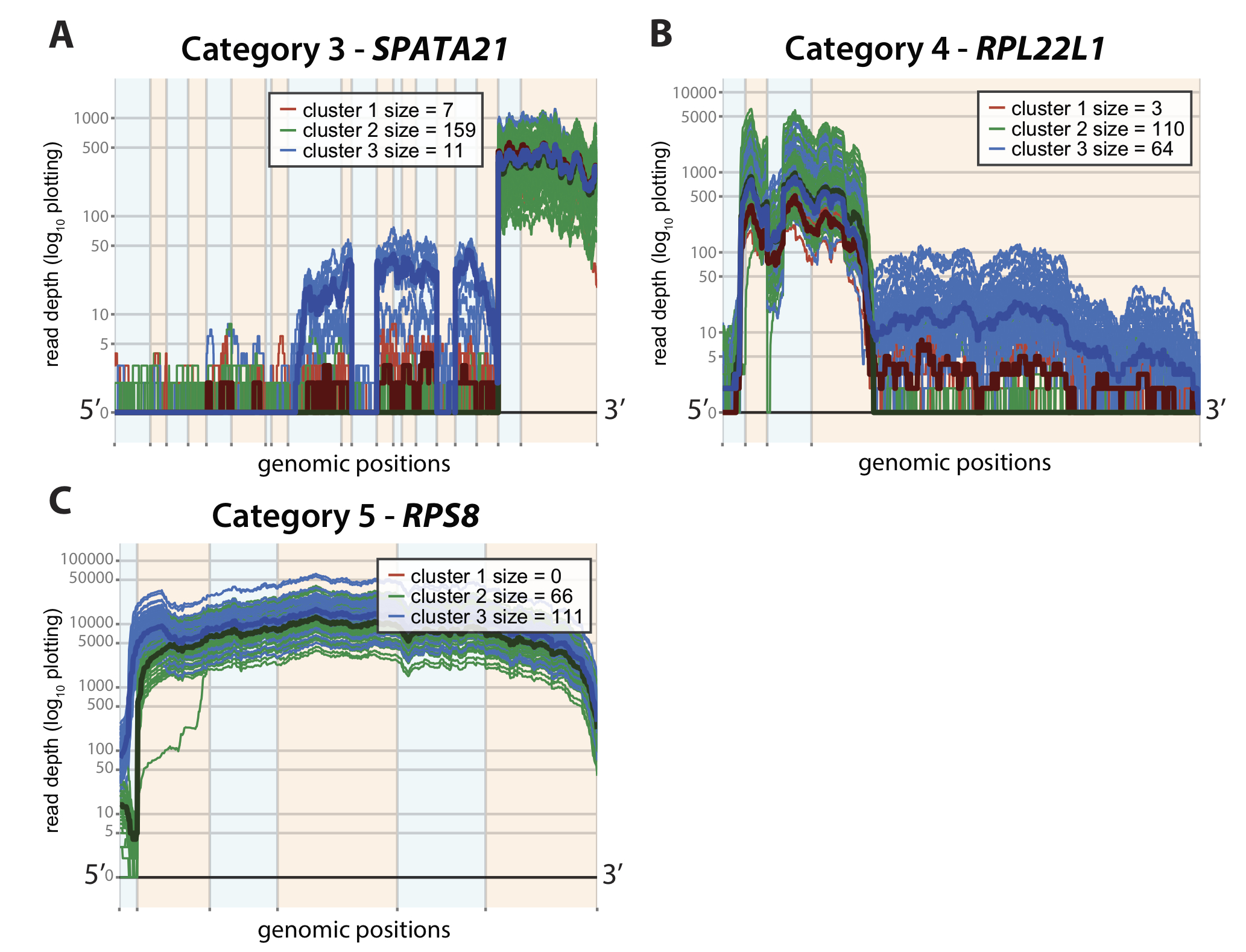


Figure S3: Representative genes for three common patterns of differential expression are shown: (A) differential usage of the 5’-end (Cat. 3), *SPATA21*, (B) differential usage of the 3’-end (Cat. 4), *RPL22L1*, and (C) differential usage of a short first exon (Cat. 5), *RPS8*. Alternating beige and blue are used to denote annotated exon boundaries. Red, blue and green represent clusters of low expression, isoform usage 1 and isoform usage 2. Bold lines denote cluster median expression.

**Supplementary Figure S4**

**PCR results**


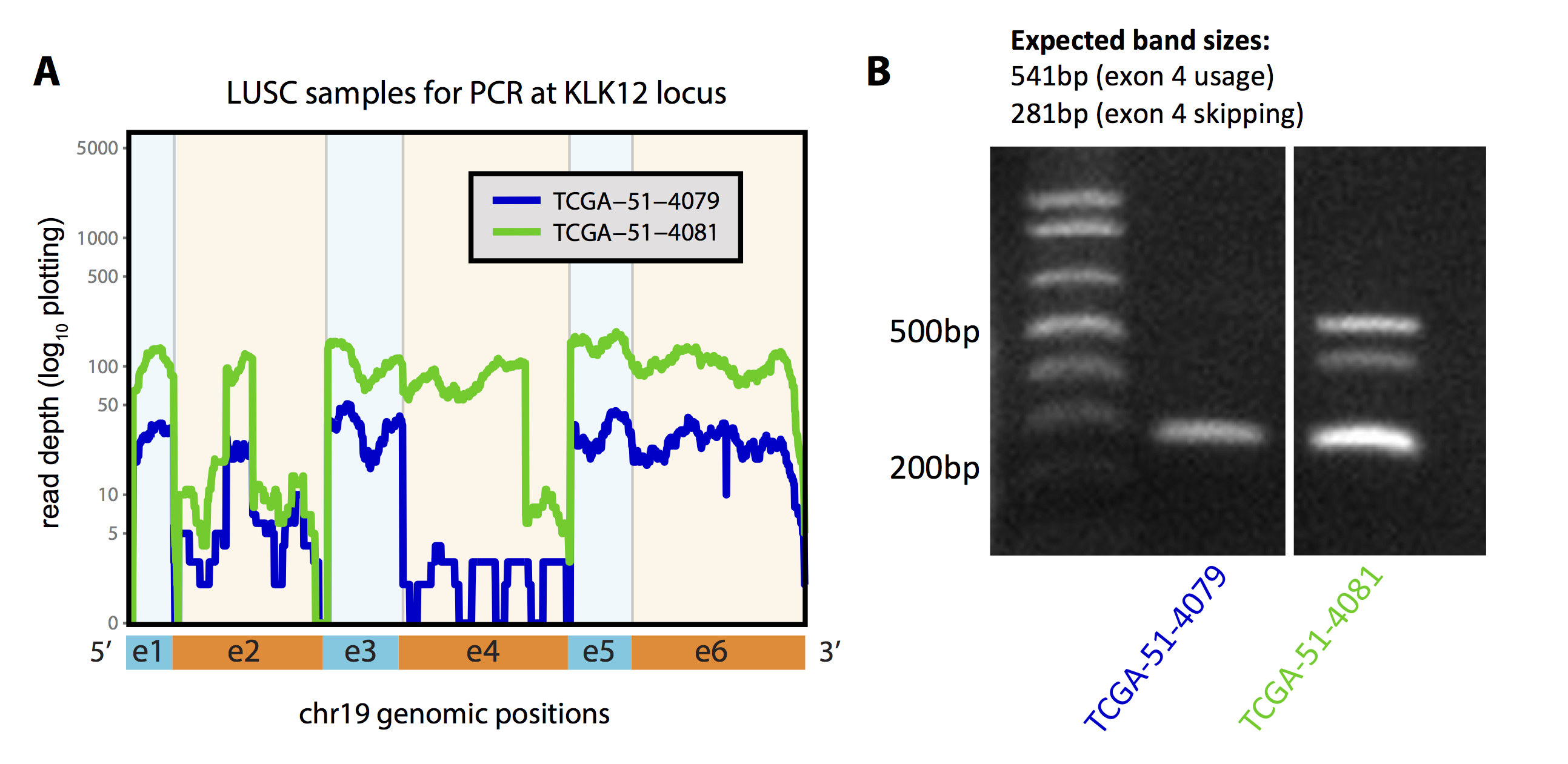


Figure S4: (A) The expression curves are shown for two samples selected for validation. Sample TCGA-51-4079 clearly shows a drop in expression at exon 4. (B) The inclusion or exclusion of exon 4 within KLK12 gene transcripts was assessed by PCR and results were visualized by agarose gel electrophoresis as described in the Methods. Primers were chosen as described in (17) such that exon 4 exclusion or inclusion corresponds to 281bp and 541bp fragments, respectively. The *KLK12* transcripts from sample TCGA-51-4079 do not contain exon 4 as the expected 541bp fragment was undetected. However, both samples show expression of the exon 4 skipping transcript, as observed by the 281bp fragment. Note that the difference between the 541bp and 281bp is the 260bp region corresponding to exon 4. The gel was performed with a third sample, which was excluded from this figure for clarity. The white space between the two lanes in (B) corresponds to where the image was modified to remove the third sample.

**Supplementary Figure S5**

**Exon analysis results**

**
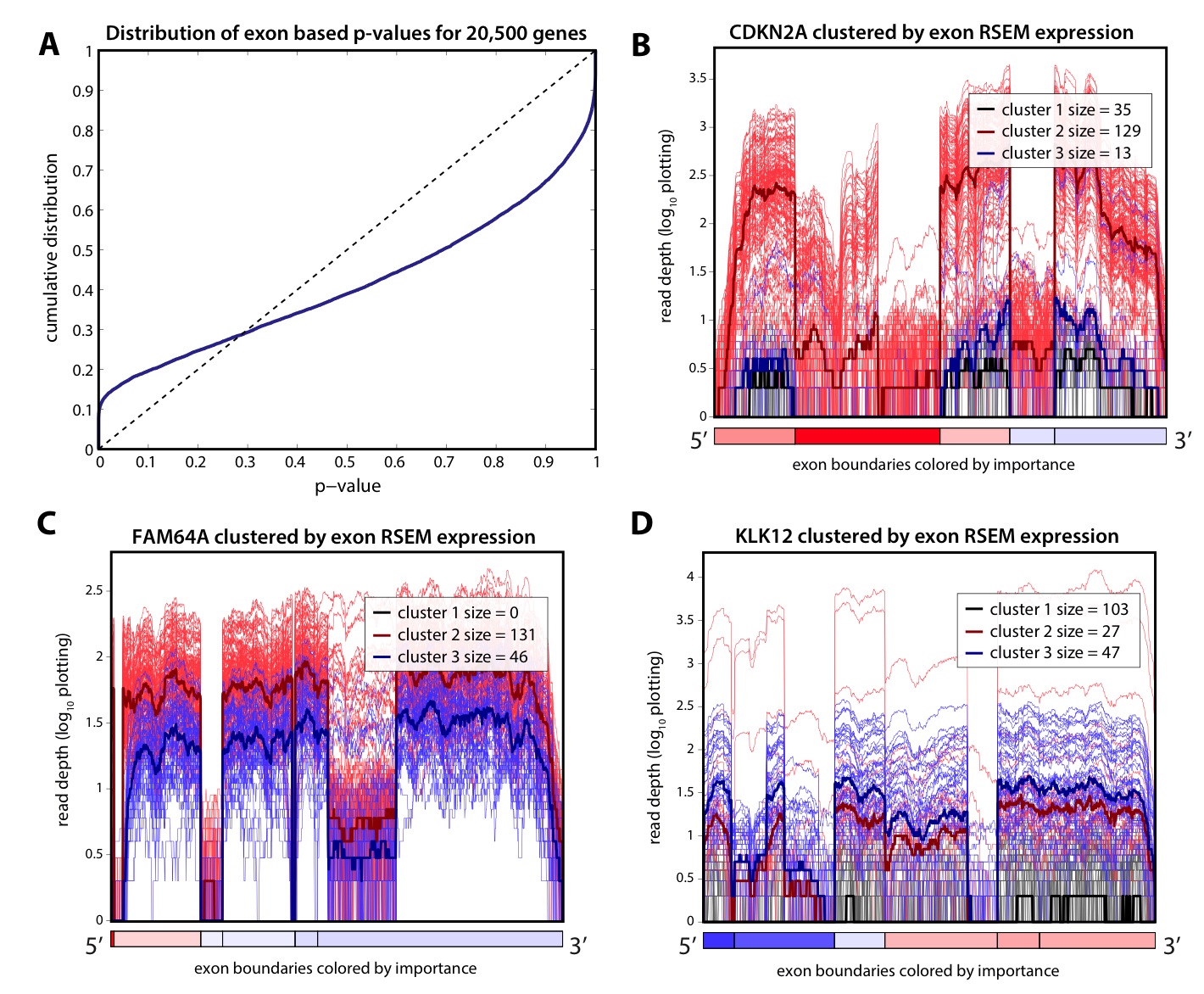
**

Figure S5: Results from applying a SigFuge type analysis to RPKM exon expression data. (A) Empirical CDF of p-values from the exon analysis. Expression curves at the (B) *CDKN2A*, (C) *FAM64A*, and (D) *KLK12* loci colored by exon-based clustering. Clusters do not capture the differential identified by the per-base analysis described in the manuscript (Figure 3). Blocks corresponding to exon boundaries are plotted below the expression curves, and colored according to their importance in separating the two clusters. That is, darker exon blocks contribute more to distinguishing the blue and red clusters, e.g. exons 1 and 2 contribute most to the clustering of *KLK12*. Red, blue and black correspond to the two expression clusters and low expression cluster. Cluster medians are plotted in bold.

**Supplementary Figure S6**

**RNA-seq simulation gene models**

****Figure S6: Two gene models used in simulation settings. (A) Three exon gene model containing a cassette exon (exon 2) spliced out from isoform 1 and only retained in isoform 2. (B) Four exon gene model containing mutually exclusive cassette exons (exons 2, 3).

**Supplementary Figure S7**

**RNA-seq simulation settings**

**
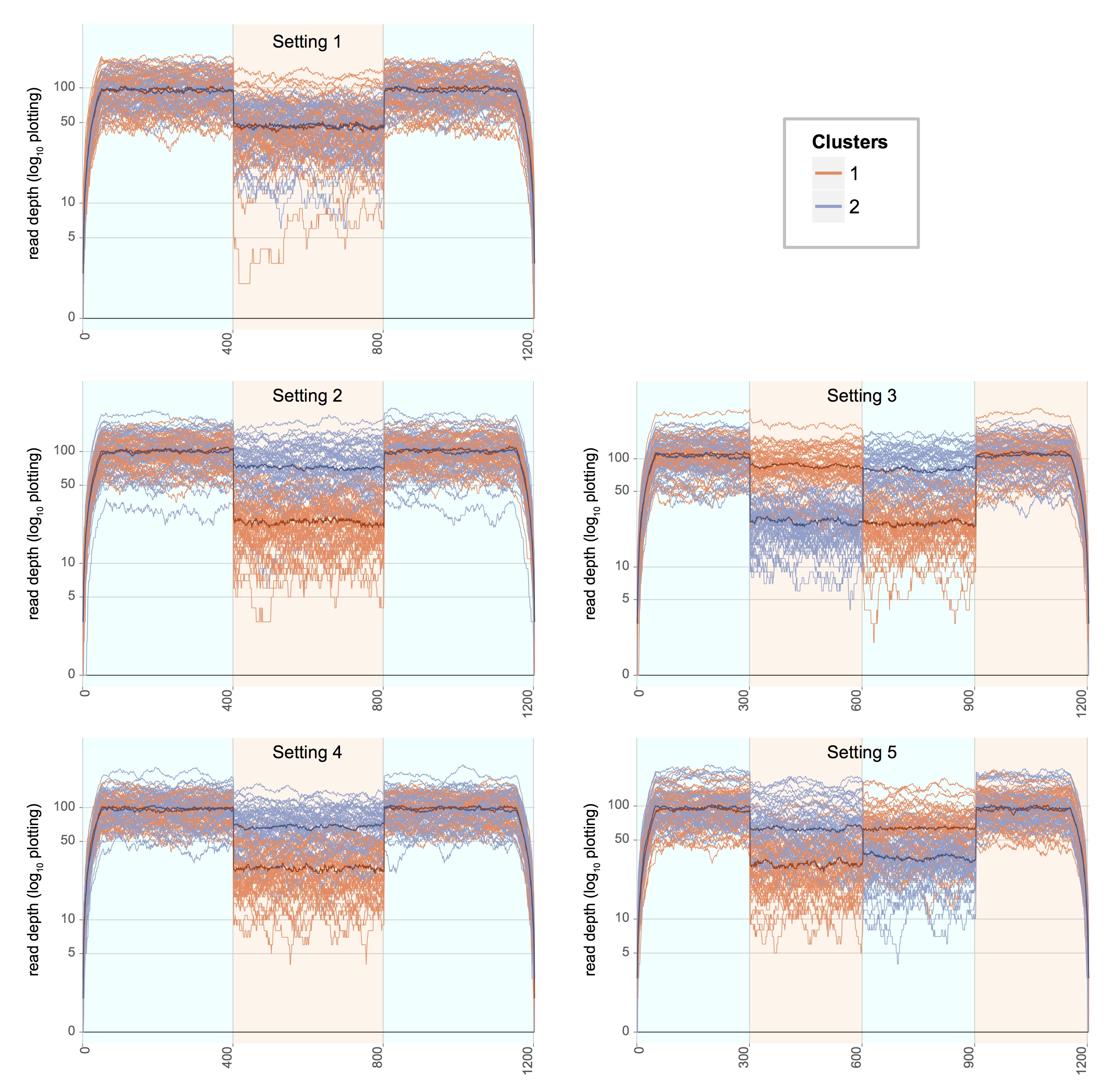
**

Figure S7: Log-transformed coverage plots are shown for 5 simulation settings. For each simulated gene locus, the per-base coverages were simulated from two underlying populations exhibiting differential isoform usage, denoted used by red and blue. Each dataset plotted was simulated with mean coverage (**μ**) 100, over-dispersion (**φ**) 0.179, gene length 1200bp, and 50 samples in each class. The population medians are shown in darker lines. Settings 1-5 are as described at the end of Supplementary Methods S2.

**Supplementary Figure S8**

**Head and neck SQCC clustering at *CDKN2A*, *FAM64A*, *KLK12***

**
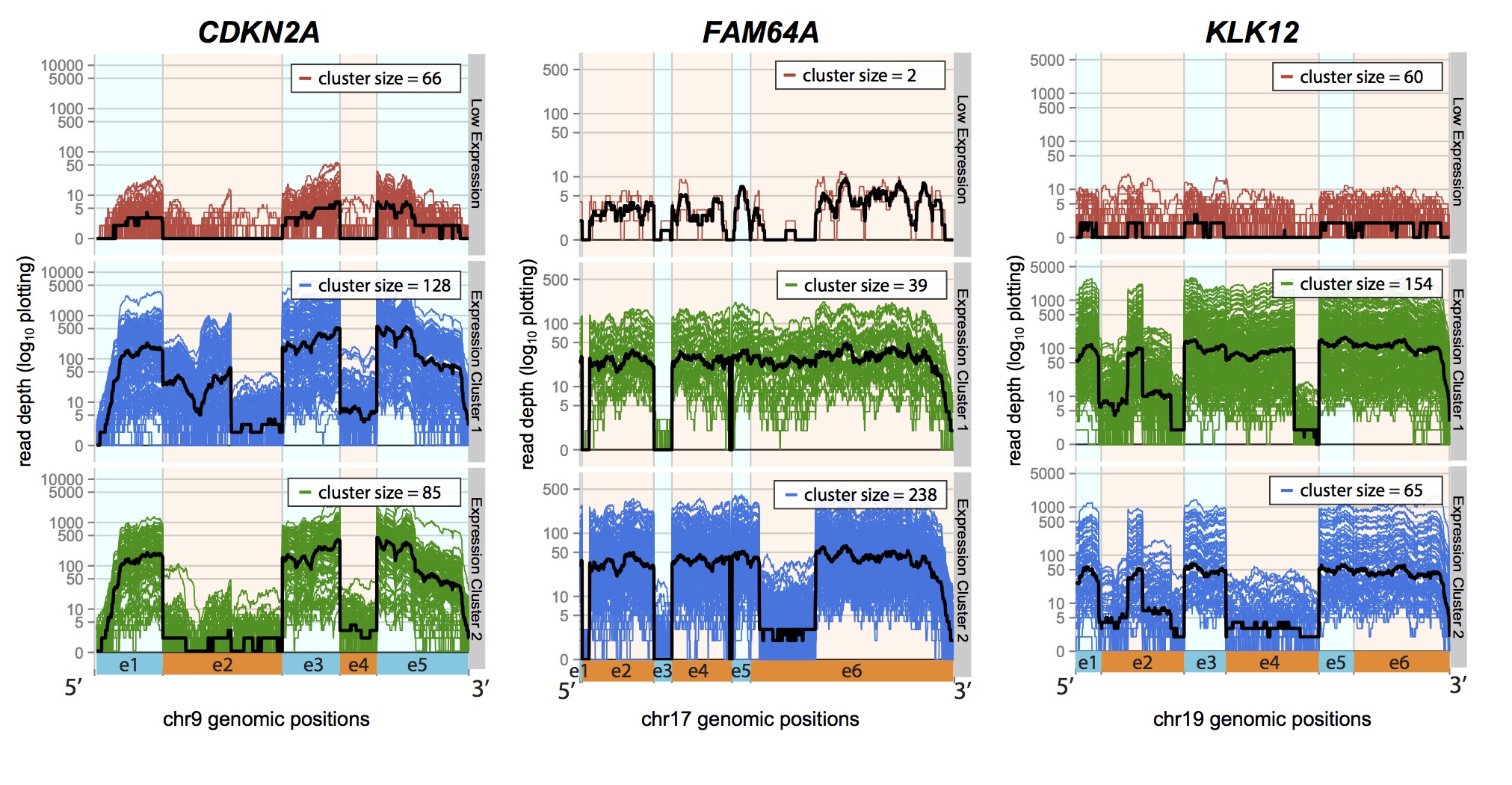
**

Figure S8: SigFuge clusters in a cohort of head and neck SQCC samples for three genes identified as highly significant in the lung SQCC samples. All three genes were found to be highly significant head and neck SQCC, with clustering patterns strongly resembling those identified in lung SQCC (Figure 2). Alternating orange and blue are used to denote annotated exon boundaries.

**Supplementary Results S1**

**Lung SQCC gene categorization**

In this section, we describe how the set of 322 significant genes were separated into Categories 1 through 6. First, expression plots, as shown in Figure 3 and Supplementary Figure S3, were created for all 322 genes. First, genes were determined to be potential artifacts if upon visual inspection, they matched any of the following criteria:

1. potential unmapped short exon: if the two expression clusters appear to only differ by overall expression level except at a short exon which has zero coverage in the cluster with lower expression.
2. potential homologous region: If at least one of the two expression clusters exhibit erratic expression patterns suggesting gapped reads misaligning to positions within the current gene and nearby homologous regions.

These criteria comprise, respectively, 65 and 26 of the 110 genes in Category 6. Next, genes were determined to fall in Categories 1-5 by the following criteria:

1. cassette exon: If the region of differential usage between the two expression clusters does not begin or end in the expressed region of the gene locus.
2. outliers: If one of the expression clusters is comprised of less than 5 samples.
3. diff. use of 5’- exons: If the region of differential usage between the two expression clusters begins at the 5’ end of the expressed region of the gene locus.
4. diff. use of 3’- exons: If the region of differential usage between the two expression clusters ends at the 3’ end of the expressed region of the gene locus.
5. alternative start sites: If the expression clusters show similar behavior as the potential unmapped short exon case described above, but with high overall coverage in all samples, e.g. ~10,000 coverage along the locus.

Genes that did not clearly meet these criteria comprise the remaining 19 genes in Category 6.**Supplementary Table S1**

**Significant genes by SigFuge analysis**

See attached Excel spreadsheet, SigFuge-supplementTables.xls.

**Supplementary Table S2**

**Significant genes by exon-based analysis**

See attached Excel spreadsheet, SigFuge-supplementTables.xls.

**Supplementary Table S3**

**Additional RNA-seq simulation results**

See attached Excel spreadsheet, SigFuge-supplementTables.xls.
